# Supplementary material for: Spatial dynamics of malaria transmission
Source: PLoS Comput Biol. 2023 Jun 12;19(6):e1010684. doi: 10.1371/journal.pcbi.1010684 (PMC10289676; doi:10.1371/journal.pcbi.1010684)
Supplement: Attachment — Submitted filename: Response2Reviewers.pdf [file pcbi.1010684.s005.pdf]

Reviewer's Responses to Questions

**Comments to the Authors:**

**Please note here if the review is uploaded as an attachment.**

Reviewer #1: This paper presents a general mathematical framework for modeling the spatial dynamics of the transmission of falciparum malaria. The paper is incredibly well written and presents the modular framework very clearly. It will be very helpful for anyone new to the malaria modeling field but also for those already in the research area wanting to expand to spatial models. I have only a couple of comments:

**Response:** Thank you for the kind comments.

• It would be good to have a sense of any other frameworks that are available and how they might compare, e.g. from Institute for Disease Modelling or other groups?

**Response:**

Malaria modeling models and software to implement such models are now routinely published in open source codebases. We cannot attempt an exhaustive overview of all malaria models. Recent reviews and critiques have been written by us and by others. The most notable examples are the suite of models from Imperial College London which have been released as the "malariasimulation" package on GitHub (https://github.com/mrc-ide/malariasimulation), the OpenMalaria suite of models developed by the Swiss Tropical and Public Health Institute also on GitHub (https://github.com/SwissTPH/openmalaria), and the EMOD modeling framework developed by the Institute for Disease Modeling (https://docs.idmod.org/projects/emod-malaria/en/2.20_a/). All three of these models are large, individual-based, stochastic simulations. They are highly detailed, can simulate a large variety of health and vector control interventions, and require large numbers of parameters to be specified regardless of the intended granularity of simulation.

Our framework takes a different approach, based on differential equations, to build models of varying granularity. Components are modular, based on matrix operations, and designed to be changed to suit specific analyses. An obvious disadvantage however, is the inability to model stochasticity in the modeled processes, which is especially relevant at small population sizes, or when the disease is near extinction.

There are other features of the framework (*e.g.* temporal dynamics) that we plan to describe in other peer reviewed publications soon, so we feel that a detailed comparison with other frameworks would be incomplete. We agree with the reviewer that this would be useful, but we feel it would be better in a future manuscript.

• Since the framework solves systems of ODEs, it would be helpful to have a sense of the contexts that this framework could apply to. Eg control, elimination, both?

**Response:** The framework is being designed as a tool for policy analytics to serve the needs of malaria control programs. We are currently writing a follow-up document that looks at the temporal dynamics of malaria that will extend the functionality to include malaria control.

Reviewer #2: This is a very well written description of a detailed, yet adaptable / scalable framework for a spatial malaria model.

The framework is well constructed and appears to be both logically and mathematically consistent and appropriate. I particularly like the very clear and logical separation of structural and dynamic components of the model as well as the explicit specification of spatial matrices. Another very nice and useful approach is the use of the reproductive number to assess the importance of different model components to transmission and their importance for predicting impact of different control measures targeting individual components.

**Response:** Thank you for the kind comments.

The challenges with this very nice conceptual framework is that although an implementation in R code, the presentation feels largely a theoretical framework rather than demonstrating its actual implementation. Some of the model components - in particular the spatial structures and the detailed vector components are very detailed and require complex parameterisation (with most parameters being vectors or matrices). Doing this in practice. Defining these parameters will require a lot of data (which may not be available for many other settings) and very extensive model fitting to work in any actual implementation of the model framework.

**Response:** We acknowledge some limitations of the software package in its present form, which is now published on CRAN as ***exDE***. We are currently working to address some of the limitations -- we are trying to make the models as easy to set up and analyze as possible.

We hasten to point out that this is a *modular* framework. Scalable complexity makes it possible to develop models of any degree of complexity -- the Ross-Macdonald model is, in fact, a special case. We also note that while models could be built that would require complex parameterization, there are many tricks to limit the number of parameters used when it comes time to fit the models to data. For example, the values of the parameters in the models could be assigned through another study using environmental covariates. The purpose of this framework was to enable development of spatial models, not to address a broader set of issues related to model complexity, computational complexity, parsimony and the way these play out in science and policy. On the other hand, we plan to address these issues in new manuscripts soon.

It would be great to see more details on how the authors implemented their framework for bioko but I assume that will happen in one or several follow-up publications.

The analysis of malaria transmission dynamics and control on Bioko Island is a work in progress. We have published some simulation modeling studies and other analyses for Bioko Island, but we have so far avoided tackling the problem of assessing the impact of malaria control on Bioko Island. We plan to do so, but we need to extend the framework to discuss malaria transmission as a changing baseline that has been modified by control. The current version of exDE on github has added functionality to describe exogenous forcing (by weather,

travel, etc.) and effect modification by vector control. The analysis of malaria transmission on Bioko Island is forthcoming.

Another minor concern is that the presented implementation of the epidemiological / within host model is simple. This is sufficient to demonstrate how the different components of the framework fit together and interact but - as the authors point out - may have to be substantially more complex for addressing specific public health questions.

**Response:** We acknowledge the limitation of the model that we presented. As the reviewer points out, the model was complex enough to illustrate the features of the framework, but it would be inadequate as a policy model. Development of exDE and its supporting model libraries is active and ongoing. One facet is development of new models for malaria epidemiology. The topic of malaria epidemiology is vast and complicated. In this manuscript, we wanted to avoid trying to cover the material from exposure through infection and immunity, parasite densities and detection, disease, infectiousness, drug taking and chemoprotection. These new models for malaria that are complex enough to work in policy, but they would not have been appropriate for this manuscript.

To address this concern, we added some text in the paragraph that follows *Core Dynamical Components*

*These particular models were chosen because they are complex enough to illustrate key features of the framework. These models might not be appropriate for some studies -- in particular, the model for epidemiology is too simple for policy. Since the framework is modular, other models can be developed that suit the needs of a study.*

Lastly, I do have some concerns in regards to who may actually be able to use the R code that is provided. It is very well written, beautiful code. However, it seems to fall a bit in between potential two main user classes. Given that for any extensions/ adaptations specific ODEs need to be specified and programmed, users that are not well familiar with writing and programming ODEs may struggle to adapt the code to their setting and problem. On the other hand highly proficient ODE programmers might find it (almost) as fast to just program their model from scratch.

**Response:** We agree that for a set of advanced users who are skilled at programming, and translating math into code, those persons may find it preferable to program their own model implementing our framework from scratch. Our opinion is that the package released with the paper should be considered a “reference implementation” of the proposed framework against which other researchers can test their implementations, if they choose to go that route. We have a large set of automated tests, including many tests that systems started at an equilibrium point remain at equilibrium (up to floating point error). These tests can become a standard against which other implementations can be evaluated to ferret out subtle bugs. Our main interest is in sharing/promoting the mathematical framework, and keeping the code as a reference implementation.

For users less proficient at programming, we hope that interested parties can contact us, preferably through GitHub. We updated the contributing section of the README

(https://github.com/dd-harp/exDE/#contributing) to be more clear that we welcome all forms of contribution regardless of experience, including questions on how to use the software, as this will help us improve our documentation. We also made it more clear that we invite interested persons to contact us directly about larger collaboration/model development, such as a new mosquito model.

However, none of these minor concerns distract from the high quality of the manuscript.

We made some minor changes to the text.

- Around line 470, we felt that a slight change would make the modularity more apparent to the reader:
  - OLD: All models developed in this framework must accept the adult emergence rates, $\Lambda$, and they must be formulated in enough detail to specify a population of egg-laying mosquitoes, G, to compute v
  - NEW: All models developed in this framework must accept the adult emergence rates, $\Lambda$, and they must be formulated in enough detail to compute the population egg-laying rate, $\Gamma$.
- Around line 1084, we deleted two words that appear to have been an editing error: We changed "through a parasite, a full parasite" to "through a full parasite"
- Around line 1105: "data … is" was changed to "data … are"
